# Carriage epidemiology of Moraxella catarrhalis in an all-age community cohort between 2016 and 2018

**DOI:** 10.1099/acmi.0.001117.v3

**Published:** 2026-06-30

**Authors:** Denise E. Morris, Alex J. J. Lister, David W. Cleary, Stuart C. Clarke

**Affiliations:** 1 Faculty of Medicine and Institute for Life Sciences, University of Southampton, Southampton, UK; 2 Department of Microbes, Infection and Microbiomes, School of Infection, Inflammation and Immunology, College of Medicine and Health, University of Birmingham, Birmingham, UK; 3 Institute of Microbiology and Infection, University of Birmingham, Birmingham, UK; 4 NIHR Southampton Biomedical Research Centre, University Hospital Southampton NHS Trust, Southampton, UK

**Keywords:** antimicrobial resistance (AMR), carriage, epidemiology, *Moraxella catarrhalis*

## Abstract

*Moraxella catarrhalis* is an increasingly important pathogen, recognized as a common cause of respiratory tract infections. It is particularly known for its role in causing otitis media in children and exacerbations of chronic obstructive pulmonary disease (COPD) in adults. With growing interest in developing vaccines against *M. catarrhalis*, a deeper understanding of epidemiology in both carriage and disease is crucial. Here, we present an all-age, community-based, upper respiratory tract carriage study (the Solent SMART Study) designed to investigate the epidemiology of, and risk factors for, *M. catarrhalis* carriage. In total, *n*=1,622 community-based participants were recruited with an additional *n*=79 individuals recruited from care/nursing homes in the Southampton/Hampshire UK region from whom a total of *n*=228 *M*. *catarrhalis* were isolated. Carriage prevalence was 8% (95% CI: 6.7–9.4%) in community-based participants, 19% (95% CI: 11.0–29.4%) in care/nursing home residents and 4.7% (95% CI: 1.6–10.7%) in the community-based subset with COPD (*n*=106). Nasopharyngeal carriage site, young age, microbial co-carriage with *Streptococcus pneumoniae*, *Haemophilus influenzae* and *Neisseria meningitidis* and recent/concurrent respiratory tract infection were all positively associated with the carriage of *M. catarrhalis*. Antimicrobial resistance testing showed that *n*=91 (41.4%) of the 220 isolates tested resistant to at least 1 antibiotic, with the most frequent being resistance to chloramphenicol (*n*=76, 34.5%) and ciprofloxacin (*n*=64, 29.1%).

**Impact Statement**

This study provides comprehensive, all-age, community-based investigation of *Moraxella catarrhalis* carriage and antimicrobial resistance (AMR) in a UK cohort. Whilst *M. catarrhalis* is recognized as an important cause of otitis media in children and exacerbations of chronic obstructive pulmonary disease in adults, data on its carriage epidemiology beyond childhood have been limited. By including both community participants and care/nursing home residents, this work adds novel insight into risk factors for colonization across the life course and highlights the higher prevalence in institutionalized older adults. The analysis of co-carriage with other respiratory pathobionts further advances understanding of microbial interactions relevant to disease and treatment outcomes. Importantly, we report AMR profiles, contributing much-needed surveillance data to inform future antimicrobial therapeutic strategies. These findings have broad relevance to infectious disease epidemiology, respiratory medicine and public health, supporting both vaccine development and antimicrobial stewardship. As a pre-pandemic dataset on *M. catarrhalis* carriage, this study provides an incremental but significant step in addressing knowledge gaps and offers a valuable baseline for monitoring post-pandemic trends and informing future interventions.

## Data Summary

The authors confirm that all supporting data, code and protocols have been provided within the article, through supplementary data files or via data repository, accessed at https://figshare.com/articles/dataset/M_catarrhalis_AMR_breakpoints_xlsx/29401931?file=55638299.

## Introduction

*Moraxella catarrhalis* is a Gram-negative diplococcus and human pathogen of increasing importance, which is now recognized as one of the most common causes of respiratory tract infections (RTIs) [1,2] specifically exacerbations of chronic obstructive pulmonary disease (COPD) [3] in adults and otitis media in children [4,5].

Increasing interest in the development of vaccines against *M. catarrhalis* necessitates a better understanding of both disease and carriage epidemiology to inform both vaccine development and implementation strategies [6]. Little has been published on the potential risk factors for *M. catarrhalis* carriage, with studies predominantly focused on disease or carriage examined primarily in young children [7] resulting in a paucity of data from teenagers and adults. In children, *M. catarrhalis* carriage is highly prevalent, up to 66% by age one and 77.5% by age two in longitudinal studies, and detection rises significantly during otitis media episodes from around 27% during healthy visits to over 60% during infection [8,9]. Understanding carriage patterns in older age groups is particularly important given their frequent exposure to healthcare settings and underlying conditions that may predispose to bacterial interactions and infection.

It is known that *M. catarrhalis* is frequently detected alongside *Streptococcus pneumoniae* and non-typeable *Haemophilus influenzae* (NTHi). This can have significant clinical implications as co-infection can result in *β*-lactamase from *M. catarrhalis* conferring protection for *S. pneumoniae* and NTHi against *β*-lactam antibiotics [10,11]. Furthermore, those with COPD are a cohort where the acquisition of *M. catarrhalis* is a risk factor for exacerbation [12]. Therefore, gaining a better understanding of the carriage of *M. catarrhalis* in this group, and the risk factors of such carriage, is vital and of clinical interest. Similarly, care/nursing home residents are a cohort with no prior data related to *M. catarrhalis*, which often suffer from COPD and frequent RTI.

Antimicrobial resistance (AMR) is an increasing global health threat, resulting in diminished therapeutic benefits and restricted treatment choices. Preliminary research has identified that *M. catarrhalis* has a variety of mechanisms which reduce antibiotic susceptibility [10]. This includes *β*-lactamase production which leads to reduced sensitivity to *β*-lactam antibiotics with an incidence of over 90% of isolates [11,13] to as much as 99% in one report from China [14]. Resistance to tetracycline has been shown in almost two thirds of isolates, also in China, with liberal use of antibiotics in clinical and community settings being highlighted as the cause [15]. Increasing resistance is a concern, as shown by the rise in minimum inhibitory concentration for several common antibiotics, including cefaclor, cefuroxime and tetracycline to name a few in a study from Taiwan [16]; thus, continued surveillance is required to help reduce the emergence of resistant strains.

*M. catarrhalis* demonstrates pronounced seasonality in respiratory carriage and infection. In children, nasopharyngeal carriage increases substantially in winter compared to summer or spring [17,18]. Adults, especially those with chronic pulmonary conditions, also experience higher rates of *M. catarrhalis* infections in winter and spring [17,19]. Smoking has also been identified as a significant risk factor for increased bacterial carriage [1,2]. Additionally, racial disparities have been studied, with evidence showing ethnic minority groups are at higher risk of pathogenic bacterial carriage [3]. However, further research is required to establish such associations for *M. catarrhalis*.

Here, we present a community-wide, all-age carriage study (the Solent SMART Study) designed to investigate the epidemiology of *M. catarrhalis*, including risk factors for carriage across two winter periods. Prevalence of AMR was also assessed. This is especially important as UK AMR data for this bacterium are limited in the published literature.

## Methods

The Solent SMART Study was a large, community-based respiratory carriage study conducted over two winter seasons (October 2016–March 2017 and October 2017–March 2018). Ethical approval was granted by the NHS Research Ethics Committee (REC 16/SS/0094) and Solent NHS Trust R and D (SR/002/16).

Participants of all ages were recruited from Solent NHS Trust sites during clinic appointments, either as attendees or accompanying individuals. Additional recruitment took place at partner locations, including care homes, nursing homes and other community settings (Appendix A). Three participant groups were defined for analysis: community-based participants, care/nursing home residents and those with COPD. Community-based participants and care/nursing home residents were treated as mutually exclusive; however, participants with COPD were part of the community cohort. Care/nursing home residents were recruited via a distinct process: a letter of invitation was sent to the home manager, and the research team attended only following managerial approval. Given the vulnerability of this population, a trained mental health and older persons' research nurse confirmed mental capacity in accordance with the Mental Capacity Act 2005 before informed consent was obtained. Care/nursing home residents were classified as a separate cohort and excluded from all community-based analyses. Participants with COPD were not separately recruited but were identified as a sub-group of community-based participants via self-report on the questionnaire. To supplement opportunistic community recruitment of COPD participants, dedicated COPD clinics and therapy sessions were attended during the recruitment period. As care/nursing home residents were excluded from community analyses, the COPD sub-group comprises community-based participants only. Whilst it is acknowledged that some care/nursing home residents may also have had COPD, these individuals were captured within the care/nursing home cohort and not the COPD sub-group.

English-language participant information sheets, tailored to different ages and literacy levels, were provided. Following an opportunity to ask questions, written informed consent was obtained in accordance with Good Clinical Practice. Parents or guardians consented for children aged 0–10, whilst participants aged 11–16 provided assent alongside parental consent. Inclusion criteria included the ability to provide informed consent or assent with parental consent. Individuals with obstructed nasal passages or unable to provide consent were excluded. To reduce potential household-level bias, only one participant per family was enrolled. This study was designed to assess asymptomatic upper respiratory tract carriage of *M. catarrhalis*. Participants were not recruited based on clinical diagnosis or symptoms of infection, and no diagnostic criteria were applied to differentiate between colonization and infection. All findings therefore relate to carriage status only.

Participants or their guardians completed a short questionnaire collecting demographic information and data on recent RTIs, antibiotic use, long-term illness, up-to-date vaccination status (as per the UK schedule) with ‘up to date’ referring to completion of all recommended, smoking status (adults only), passive smoke exposure and day-care attendance (young children only). Long-term illness was defined as a health problem that is expected to last for a year or longer and cannot be cured, and examples include asthma, COPD, diabetes and epilepsy. Recent RTI was defined as having a self-reported RTI within the last month at the date of completion of the questionnaire. No further details were requested on the causative agent of the infection. Recent antibiotic use was defined as being prescribed and taking an antibiotic within the last month at the date of questionnaire completion. Ages 0–10 and 11–16 years were asked if they received the pneumococcal conjugate vaccine (PCV) [Prevenar13, (PCV13)]. At the time of the study, PCV13 was the only PCV available in the UK. The national childhood immunization schedule followed a 2+1 regime. Ages 17–49 and 50+ were asked if they had received PPV23 pneumococcal vaccine.

Each participant was asked to provide three samples: nasopharyngeal, oropharyngeal and nasal swabs. All samples were taken by trained members of the research team. Swabs were cultured on a combination of general-purpose and selective media, including Columbia Blood Agar with horse blood (CBA, Oxoid PB0122), Columbia Blood Agar with chocolated horse blood (CHOC, Oxoid PB0124), Columbia Agar with colistin and nalidixic acid (CNA, Oxoid PB0308), Columbia Agar with chocolated horse blood and bacitracin (BACH, Oxoid PB0220) and lysed GC selective agar (GC, Oxoid PB0962). All plates were incubated at 37 °C in 5% CO₂ for 24–48 h. Each target organism was identified using a sequential approach combining selective culture, colony morphology, Gram staining and targeted confirmatory biochemical tests, each chosen for their established ability to discriminate against phenotypically similar species likely to be encountered at the sampled anatomical sites.

*M. catarrhalis* was initially identified by colony morphology: non-haemolytic, opaque, flat colonies that remained intact when pushed across the agar surface, typically appearing grey-white on blood agar or pinkish-brown on CHOC plates. Suspected isolates were confirmed as Gram-negative and oxidase-positive. Tributyrin hydrolysis (Sigma-Aldrich 75744-300) was then performed to differentiate *Moraxella* spp. from phenotypically similar *Neisseria* spp., which test negative for tributyrin hydrolysis. DNase testing (VWR EOLAPP0560) was subsequently used to confirm *M. catarrhalis* specifically, as it is DNase-positive, whereas other *Moraxella* spp. are negative [20,24]. DNase testing was incorporated following a PHE protocol update prompted by documented misidentification of *N. meningitidis* as *M. catarrhalis* in a prior carriage study, providing an additional layer of specificity beyond oxidase and tributyrin testing alone.

*S. pneumoniae* colonies appeared *α*-haemolytic, typically 1–2 mm in diameter with a draughtsman or blood-cell-like morphology, with some serotypes appearing mucoid. Optochin discs (5 µg, Oxoid DD0001) were applied to CNA plates; sensitivity to optochin (inhibition zone ≥14 mm) was used to discriminate *S. pneumoniae* from other *α*-haemolytic streptococci, including *Streptococcus mitis* and *Streptococcus oralis*, which are optochin-resistant. Suspected isolates were confirmed as Gram-positive diplococci [25]. The use of selective CNA agar, which inhibits Gram-negative organisms via colistin and nalidixic acid, further limited the range of organisms requiring exclusion.

*H. influenzae* colonies were small, round, grey and oxidase-positive, identified on selective BACH agar. Final confirmation involved X, V and XV factor disc testing on Blood Agar Base (Oxoid PO1046). Growth exclusively around the XV disc, confirming obligate dependence on both haemin (X factor) and NAD (V factor), is the standard criterion for *H. influenzae* identification and excludes other *Haemophilus* spp. that require only one factor, such as *Haemophilus parainfluenzae* (V factor only). *Haemophilus haemolyticus*, which shares the same factor requirements as *H. influenzae*, was considered as a potential confounder; however, it characteristically produces *β*-haemolysis on blood agar, which was not observed in any confirmed isolates [26].

*N. meningitidis* presented as smooth, moist, grey-brown colonies on selective GC agar, confirmed as Gram-negative cocci and oxidase-positive [27]. Final confirmation was achieved using API NH strips (bioMérieux 10400), a validated commercial biochemical identification system covering 12 reactions that reliably differentiates *N. meningitidis* from other *Neisseria* spp. and *Haemophilus* spp., following the manufacturer’s instructions.

*Staphylococcus aureus* was identified as opaque, golden colonies on selective CNA agar, which suppresses Gram-negative organisms, and confirmed as Gram-positive cocci. Coagulase positivity was determined using the Pastorex Staph Plus Kit (Bio-Rad 56353), with agglutination confirming the presence of bound coagulase, protein A and clumping factor. Coagulase positivity specifically excludes coagulase-negative staphylococci, which may produce similar colony morphology on CNA but are coagulase-negative [28].

All *M. catarrhalis* isolates were tested for phenotypic antibiotic resistance using the disc diffusion method on Mueller–Hinton F agar (Mueller–Hinton agar with 5% defibrinated horse blood and 20 mg l^−1^ *β*-NAD; Oxoid PB1229A), following EUCAST version 14.0 guidelines. Isolates were grown on Columbia Blood Agar (Oxoid PB0122), and colonies were suspended in saline to a 0.5 McFarland standard. A sterile swab was used to evenly spread the suspension across the agar surface. Four antibiotic discs were applied per plate, and plates were incubated at 37 °C in 5% CO₂ for 18±2 h. Inhibition zones were measured and interpreted using EUCAST breakpoints. The antibiotics tested included amoxicillin-clavulanic acid 2/1 µg (Oxoid CT0538B), cefotaxime 5 µg (CT0407B), ceftriaxone 30 µg (CT0417B), erythromycin 15 µg (CT0020B), tetracycline 30 µg (CT0054B), ciprofloxacin 5 µg (CT0425B), chloramphenicol 30 µg (CT0013B) and meropenem 10 µg (CT0774B). *H. influenzae* ATCC49766 was used as the control strain. Chloramphenicol was included despite not being a first-line antibiotic due to its history in AMR surveillance studies and to aid in determining the broadness of the resistance spectrum.

Swab positivity was calculated for each anatomical site. ‘True’ carriage, a Boolean variable defined by whether a person carries a bacterium or not regardless of the site or number of sites of carriage, was also determined. Prevalence of *M. catarrhalis* was analysed overall and by anatomical site and age group.

Multivariate logistic regression was conducted using SPSS version 28 to evaluate associations between *M. catarrhalis* carriage (dependent variable) and independent variables, including age, sex, ethnicity, recent RTI and recent antibiotic use. Adjusted odds ratios (ORs) with 95% confidence intervals were reported. Statistical significance was set at *P*<0.05. Given the known correlation between certain predictor variables, particularly COPD status and flu vaccination receipt, the potential for collinearity was considered in the interpretation of model estimates. Variables producing implausibly large ORs with very wide confidence intervals were flagged as likely unstable, and findings are reported with appropriate caveats.

## Results

### Recruitment

A total of 1,701 individuals were recruited, comprising 1,622 community-based participants (median age: 31 years; IQR: 46) and 79 care/nursing home residents (median age: 87 years; IQR: 14). Participant characteristics are summarized in Table 1.

**Table 1.** Participant characteristics.

		Total recruitment	
		Community-based population n (%)	Care/nursing homes n (%)
**Total study recruitment**		1,622	79
**Age**	Mean	35.1	84.1
	Min	0.02	44
	Max	94	100
	Median	32	87
	IQR	46	14
	0–4	200 (12.3)	0 (0)
	5–16	341 (21.0)	0 (0)
	17–49	540 (33.3)	1 (1.3)
	50+	541 (33.4)	77 (97.5)
	Unknown	0	1 (1.3)
**Sex**	Male	739 (45.6)	26 (32.9)
	Female	871 (53.7)	53 (67.1)
	Unknown/missing	12 (0.7)	0 (0)
**Recent antibiotic use**	Yes	223 (13.8)	27 (34.2)
	No	1,394 (85.9)	49 (62.0)
	Unknown/missing	5 (0.3)	3 (3.8)
**Recent RTI**	Yes	705 (43.5)	25 (31.6)
	No	907 (55.9)	53 (67.1)
	Unknown/missing	10 (0.6)	1 (1.3)
**Vaccination status^‡^**	Up to date	1,437 (88.6)	51 (64.6)
	Not up to date	68 (4.2)	7 (8.9)
	Unknown/missing	117 (7.2)	21 (26.6)
**Received annual flu vaccination**	Yes	661 (40.8)	50 (63.3)
	No	875 (53.9)	23 (29.1)
	Unknown/missing	86 (5.3)	3 (3.8)
**Smoker (17 year olds+ only**)	Cigarette/cigar only	164 (15.2)	4 (5.1)
	E-cigarette only	38 (3.5)	0 (0)
	Cigarette/cigar and E-cigarette	35 (3.2)	2 (2.5)
	Nonsmoker	828 (76.7)	73 (92.4)
	Unknown/missing	15 (1.4)	0 (0)
**Long-term illness**	Yes	560 (34.5)	57 (72.1)
	No	1,032 (63.6)	19 (24.1)
	Unknown/missing	30 (1.9)	3 (3.8)
**Ethnicity^§^**	African	32 (2)	0 (0)
	American	12 (0.7)	0 (0)
	South-East Asian	60 (3.7)	0 (0)
	European	1,375 (84.8)*	79 (100)^†^
	Eastern Mediterranean	34 (2.1)	0 (0)
	Western Pacific	9 (0.6)	0 (0)
	Mixed	70 (4.3)	0 (0)
	Unknown/missing	30 (1.8)	0 (0)

*93.7% of which were white British.

†100% of which were white British.

‡in line with the UK immunization schedule.

§WHO-defined regions.

**Fig. 1.**
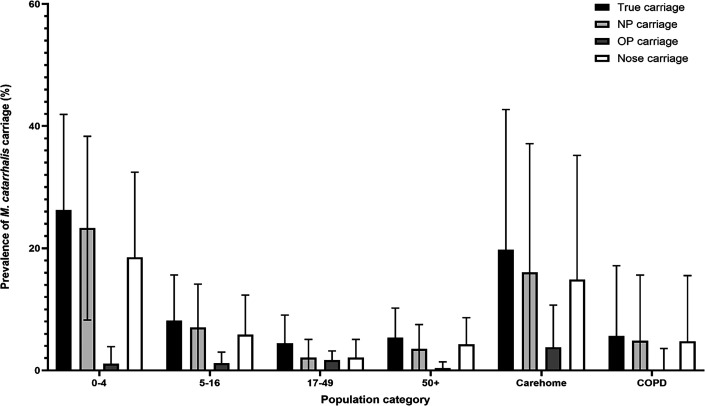
True carriage prevalence and carriage prevalence of *M. catarrhalis* in the nasopharynx (NP), oropharynx (OP) and nose by cohort.

Overall, *M. catarrhalis* was isolated from 228 participants. The ‘true’ carriage prevalence among community-based participants was 8.0% (*n*=130/1,622; 95% CI: 6.74–9.44%), whilst care/nursing home residents showed a higher prevalence of 19.0% (*n*=15/79; 95% CI: 11.0–29.4%). Carriage prevalence by age and swab site is illustrated in Fig. 1.

### COPD cohort

In total, there were *n*=106 participants with COPD (self-confirmed via the questionnaire), with an age range of 39–90 years (mean 70.5, median 71.5, IQR 66–76). The majority, 99.1% (*n*=105), of this cohort were white British, and 0.9% (*n*=1) did not provide ethnicity. In total, 49.1% (*n*=52) were female, 50.9% (*n*=54) were male and 98.1% (*n*=104) had received the annual flu vaccine. Those with COPD were recruited across two winter periods: October 2016 to March 2017 and October 2017 to March 2018 (the same recruitment period as all other participants).

Participants with COPD had a ‘true’ carriage prevalence of 4.7% (CI: 1.6–10.7). Carriage prevalence of *M. catarrhalis* was 3.9% (*n*=4/103; 95% CI: 1.07–9.65%) in the nasopharynx, 0.0% (*n*=0/105; 95% CI: 0.0–3.45%) in the oropharynx and 3.8% (*n*=4/105; 95% CI: 1.05–9.47%) in the nose. In comparison with participants of the same age range without COPD (self-confirmed via the questionnaire), the non-COPD cohort had a prevalence of 2.5% (*n*=24/965; 95% CI: 1.60–3.68) in the nasopharynx, 1.2% (*n*=11/949; 95% CI: 0.58–2.06) in the oropharynx and 2.9% (*n*=28/964; 95% CI: 1.94–4.17) in the nose. The multivariate analysis gave an OR of 1.175 (95% CI: 0.578–2.388, *P*=0.655) suggesting a non-significant trend towards increased odds of *M. catarrhalis* carriage in individuals with COPD compared to those without. Therefore, there was no significant association between COPD status and *M. catarrhalis* carriage. It should be noted that 98.1% of COPD participants had received the annual flu vaccine, compared to 40.8% of the overall community cohort. This near-complete overlap meant that COPD status and flu vaccination could not be treated as fully independent variables within the same model, and the COPD OR should be interpreted with this constraint in mind.

### *M. catarrhalis* co-colonization with pathobionts

**Fig. 2.**
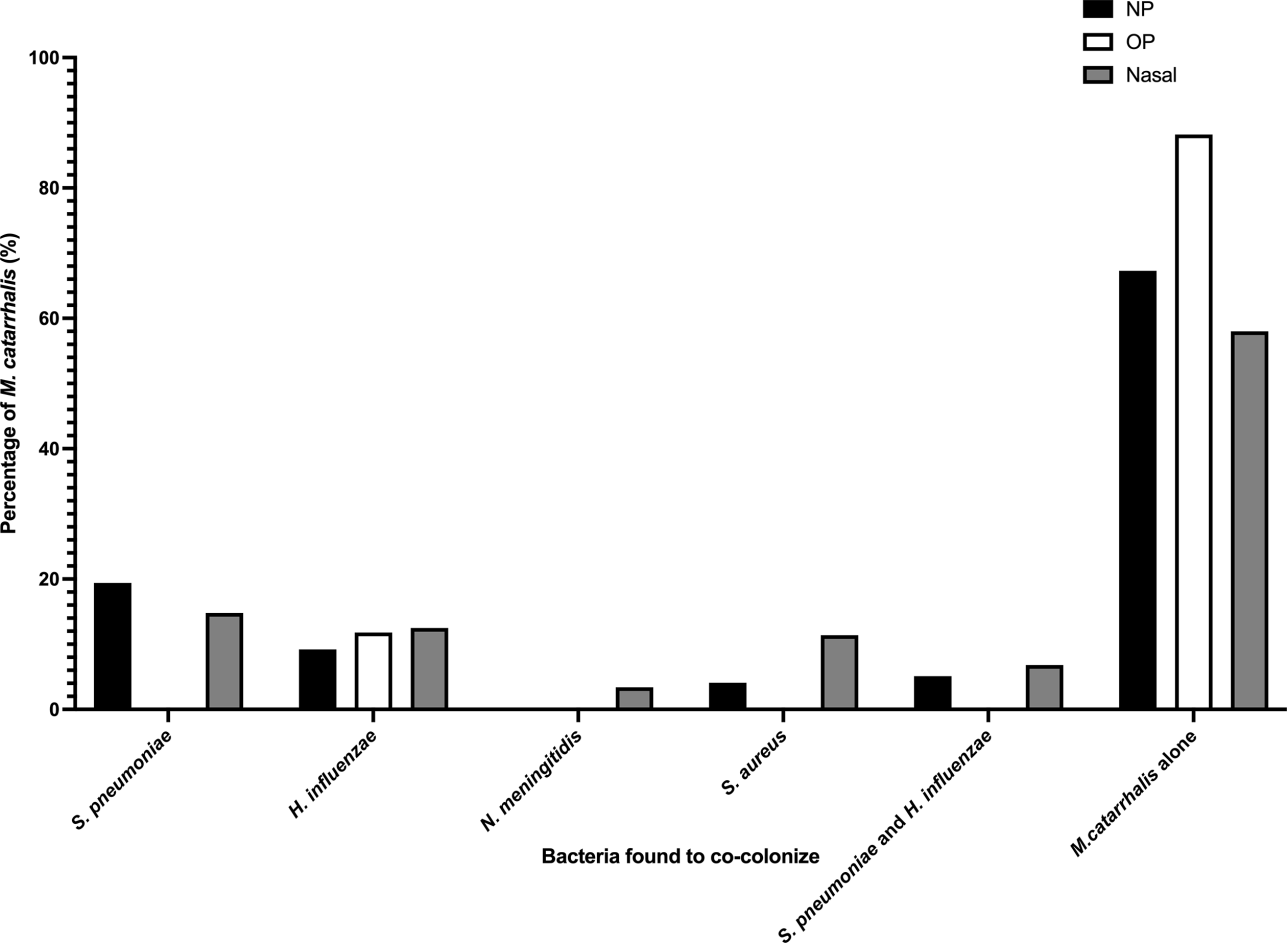
Co-colonization of *M. catarrhalis* with other bacterial pathobionts in the community.

Investigation into the carriage of *M. catarrhalis* alongside other pathobionts showed that only 3.9% (*n*=1/26; 95% CI: 0.10–19.64) of the isolates obtained from care home residents were found to co-colonize with other pathobionts, compared to 27.7% (*n*=56/202; 95% CI: 21.67–34.44) of community isolates. The single co-colonizing *M. catarrhalis* isolated from care/nursing home residents was found to co-colonize with *S. aureus*. The bacteria most commonly found to co-colonize with *M. catarrhalis* isolated from community-based participants were *S. pneumoniae* and *H. influenzae* (Fig. 2). No statistical analysis was undertaken for care/nursing home co-colonization due to insufficient data.

### Risk factors

Multivariate logistic regression was conducted to identify factors independently associated with the carriage of *M. catarrhalis*. The final model included demographic variables, microbial co-carriage, vaccination status, recent respiratory illness and exposure history. ORs and 95% confidence intervals were calculated, with statistical significance defined as *P*=<0.05. Several predictors included in the model, notably COPD status, annual flu vaccination, long-term illness and older age, are correlated in this cohort. Where such collinearity exists, individual ORs reflect the model’s partitioning of shared variance and should not be interpreted as entirely independent effects. This is particularly relevant for flu vaccination, the majority of recipients of which in this cohort were older adults or those with COPD.

When examining the site of carriage, *M. catarrhalis* was most frequently found in the nasopharynx, with a prevalence of 6.1% (*n*=98/1,622 participants; 95% CI: 5.0–7.3%). It was also found in the nose of 5.2% of participants (*n*=88/1,622; 95% CI: 2.8–6.4%) and the oropharynx of 1.1% of participants (*n*=18/1,622; 95% CI: 0.6–1.7%) in the community-based population. *M. catarrhalis* carriage at multiple sites was common, with 54.2% (*n*=71/131 participants; 95% CI: 45.27–62.93) carrying *M. catarrhalis* at more than one site. Swab site was strongly associated with *M. catarrhalis* carriage. Compared to nasopharyngeal samples, oropharyngeal swabs were significantly less likely to yield *M. catarrhalis* (OR=0.149, 95% CI: 0.090–0.246, *P*=<0.001). No significant difference was observed for nasal swabs (OR=0.924, 95% CI: 0.685–1.246, *P*=0.603).

Multivariate logistic regression showed age to be a strong predictor of carriage. Participants aged 0–4 years had the highest odds of carriage. All older age groups had significantly lower odds. The age range of care/nursing home residents was 44–100 years; however, only two participants were under the age of 60 years; therefore, a comparator cohort of all community-based participants aged 60+ years was used for further analysis. Care/nursing home residents had a carriage prevalence of 15.2% (*n*=12/79; 95% CI: 8.10–25.03) in the nasopharynx, 3.8% (*n*=3/79; 95% CI: 0.79–10.70) in the oropharynx and 13.9% (*n*=11/79; 95% CI: 7.16–23.55) in the nose. Conversely, community-based participants of a comparable age (those aged 60+, mean 71, median 70, IQR 65–76) had a carriage prevalence of 3.9% (*n*=14/360; 95% CI: 2.14–6.44%) in the nasopharynx, 0.6% (*n*=2/356; 95% CI: 0.07–2.01) in the oropharynx and 4.2% (*n*=15/360; 95% CI: 2.35–6.78) in the nose. The multivariate analysis showed no significant difference between carriage and the care home cohort.

Co-carriage of *S. pneumoniae*, *H. influenzae* and *N. meningitidis* was associated with increased odds of *M. catarrhalis* carriage (OR=2.322, 95% CI: 1.402–3.846, *P*=0.001; OR=1.366, 95% CI: 0.757–2.467, *P*=0.001; OR=5.378, 95% CI: 1.433–20.186, *P*=0.013, respectively). *S. aureus* carriage was negatively associated (OR=0.398, 95% CI: 0.225–0.702, *P*=0.001).

Vaccination status showed limited associations. Receipt of a pneumococcal vaccine was not significantly associated with carriage (OR=0.854, 95% CI: 0.551–1.325, *P*=0.481). Similarly, MenB and influenza vaccination status showed no statistical association with carriage (OR=0.876, 95% CI: 0.500–1.533, *P*=0.642; OR=1.291, 95% CI: 0.902–1.850, *P*=0.163, respectively). Notably, uncertainty or non-response often produced uninterpretable or unstable estimates, likely due to the sparsity of definitive vaccination status.

Recent cold symptoms were associated with increased odds of *M. catarrhalis* carriage (OR=3.712, 95% CI: 1.359–10.141, *P*=0.011). Recent antibiotic use showed a negative association (OR=0.715, 95% CI: 0.425–1.204, *P*=0.208), although this was not statistically significant. When examined across age groups, a divergent pattern was observed. In children aged 0–4 years, those who had recently received antibiotics showed higher *M. catarrhalis* carriage in the nasopharynx (29.4%, 95% CI: 10.3–56.0%) and nose (29.4%, 95% CI: 10.3–56.0%) compared to those without recent antibiotic use (NP: 22.4%, 95% CI: 16.6–29.1%; nasal: 17.1%, 95% CI: 11.9–23.4%), although this difference was not statistically significant (*P*=0.289, OR=1.566). In contrast, among adults aged 17–49, no *M. catarrhalis* carriage was detected in those with recent antibiotic use, compared to 2.4% (95% CI: 1.2–4.2%) nasopharyngeal and 2.4% (95% CI: 1.2–4.3%) nasal carriage in those without. Similarly, in those aged 50 and over, carriage in recent antibiotic users was 1.9% (95% CI: 0.2–6.7%) in the nasopharynx and 4.7% (95% CI: 1.5–10.6%) in the nose, compared to 3.5% (95% CI: 2.0–5.7%) and 3.7% (95% CI: 2.1–6.0%), respectively, in non-users.

Ethnicity: Compared to individuals of African origin, all ethnicities but Western Pacific had reduced odds of *M. catarrhalis* carriage. Western Pacific ethnicity was associated with a three times increased risk (OR=3.210, 95% CI: 0.708–14.549, *P*=0.130). However, none of the ORs were statistically significant.

Sex: Females had reduced odds of *M. catarrhalis* carriage compared to males (OR=0.877, 95% CI: 0.654–1.177, *P*=0.384), though this was not statistically significant.

Passive smoke exposure: Daily exposure to passive cigarette/cigar smoke was associated with lower odds of carriage (OR=0.543, 95% CI: 0.258–1.143), although this did not reach statistical significance (*P*=0.108).

Nursery attendance: Children currently attending nursery showed increased odds of carriage (OR=1.837, 95% CI: 0.937–3.600), but this did not achieve significance (*P*=0.076).

Care/nursing home residency: Sub-analysis was undertaken to investigate whether the high carriage of *M. catarrhalis* in care/nursing home residents was due to the higher age distribution of residents or the home environment itself. χ2 showed that care/nursing home residency was significantly associated with *M. catarrhalis* carriage (*P*=<0.001; OR 4.440, 95% CI: 2.31–8.52) when carriage in care/nursing home residents was directly compared to carriage in community participants of a comparable age.

As community-based participants aged 75 and over (mean 81.18, median 80, IQR 77–84) could arguably be described as more similar to the care/nursing home population, this community age range was also compared to the care/nursing home population. Again, care/nursing home residency was shown to be significantly associated with *M. catarrhalis* carriage (*P*=<0.001; OR 5.221, 95% CI: 3.03–9.00).

### Antimicrobial resistance

**Fig. 3.**
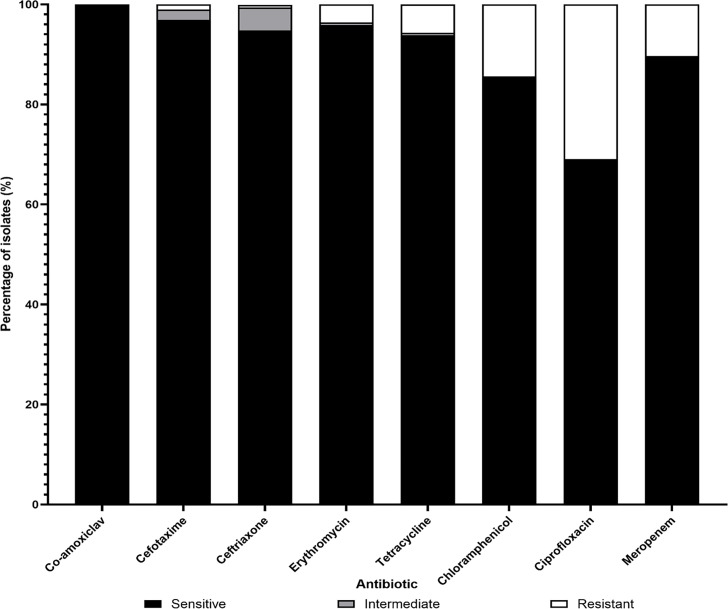
AMR of *M. catarrhalis* isolated from community-based participants. Definitions for susceptible, intermediate and resistant were based on the EUCAST version 14 definitions of susceptibility testing.

Overall, 41.4% (*n*=91/220; 95% CI: 34.78–48.18) of isolates were resistant to at least one antibiotic, and 22.7% (*n*=50/220; 95% CI: 17.36–28.84) of isolates were resistant to at least two antibiotics. Multidrug resistance, defined as resistance to three or more antibiotics, was observed in 14.6% (*n*=32/220; 95% CI: 10.17–19.91) of isolates. All isolates were susceptible to at least one of the antibiotics tested. All isolate resistance percentages can be seen in Fig. 3.

**Fig. 4.**
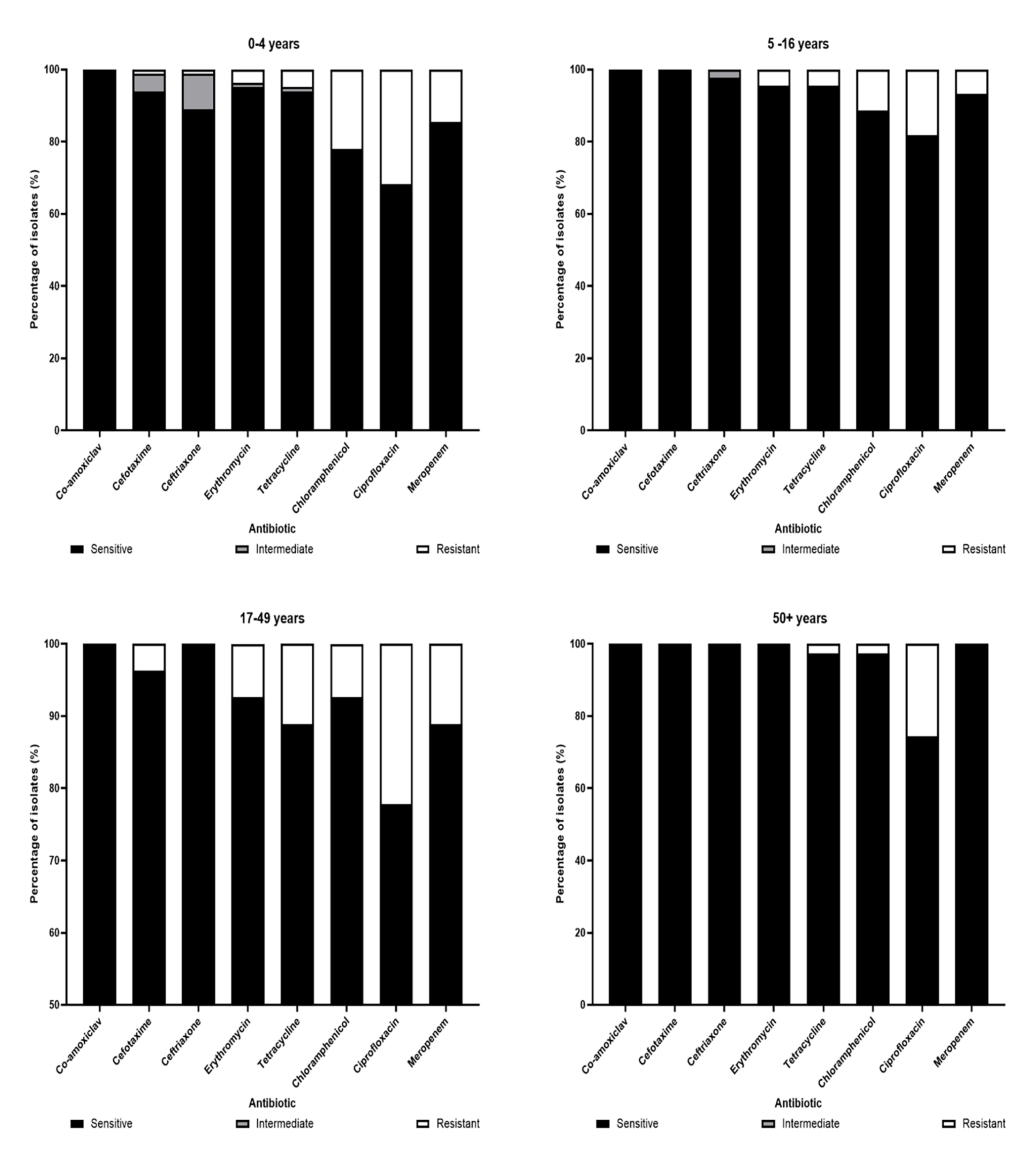
AMR of *M. catarrhalis* isolated from community-based participants by age category. Definitions for susceptible, intermediate and resistant were based on the EUCAST version 15 definitions of susceptibility testing.

In community-based participants, most resistance was seen for chloramphenicol with 34.5% (*n*=67/194; 95% CI: 27.87–41.68), then ciprofloxacin with 27.8% (*n*=54/194; 95% CI: 21.65–34.71) of isolates being resistant. In total 10.8% (*n*=21/194; 95% CI: 6.83–16.07) were resistant to tetracycline, 9.8% (*n*=19/194; 95% CI: 6.00–14.87) were resistant to meropenem, 7.2% (*n*=14/194; 95% CI: 4.00–11.81) were resistant to erythromycin, 1% (*n*=2/194; 95% CI: 0.13–3.67) were resistant to cefotaxime and 0.5% (*n*=1/194; 95% CI: 0.01–2.84) were resistant to ceftriaxone. None of the isolates were resistant to co-amoxiclav. AMR for community participants split by age group can be found in Fig. 4.

In children aged 0–4 years, resistance was observed to cefotaxime in 1.2% (*n*=1/82; 95% CI: 0.003–6.61), ceftriaxone in 1.2% (*n*=1/82; 95% CI: 0.003–6.61), erythromycin in 12.2% (*n*=10/82; 95% CI: 6.01–21.29), tetracycline in 14.6% (*n*=12/82; 95% CI: 7.80–24.17), chloramphenicol in 42.7% (*n*=35/82; 95% CI: 31.82–54.10), ciprofloxacin in 32.9% (*n*=27/82; 95% CI: 22.94–44.19) and meropenem in 14.6% (*n*=12/82; 95% CI: 7.80–24.17).

Among those aged 5–16 years, resistance to cefotaxime was 0.0% (*n*=0/44; 95% CI: 0.00–8.04), ceftriaxone 0.0% (*n*=0/44; 95% CI: 0.00–8.04), erythromycin 4.5% (*n*=2/44; 95% CI: 0.56–15.47), tetracycline 9.1% (*n*=4/44; 95% CI: 2.53–21.67), chloramphenicol 31.8% (*n*=14/44; 95% CI: 18.61–47.58), ciprofloxacin 18.2% (*n*=8/44; 95% CI: 8.19–32.71) and meropenem 9.1% (*n*=4/44; 95% CI: 2.53–21.67).

In adults aged 17–49 years, resistance was found to cefotaxime in 3.7% (*n*=1/27; 95% CI: 0.09–18.97), ceftriaxone in 0.0% (*n*=0/27; 95% CI: 0.00–12.77), erythromycin in 7.4% (*n*=2/27; 95% CI: 0.91–24.29), tetracycline in 14.8% (*n*=4/27; 95% CI: 4.19–33.73), chloramphenicol in 33.3% (*n*=9/27; 95% CI: 16.52–53.96), ciprofloxacin in 22.2% (*n*=6/27; 95% CI: 8.62–42.26) and meropenem in 11.1% (*n*=3/27; 95% CI: 2.35–29.16).

Finally, among those aged 50 years and above, resistance to cefotaxime, ceftriaxone and erythromycin was 0.0% (*n*=0/39; 95% CI: 0.00–9.03). Resistance to tetracycline was 2.6% (*n*=1/39; 95% CI: 0.06–13.48), chloramphenicol 23.1% (*n*=9/39; 95% CI: 11.13–39.33), ciprofloxacin 30.8% (*n*=12/39; 95% CI: 17.02–47.57) and meropenem 0.0% (*n*=0/39; 95% CI: 0.00–9.03).

*M. catarrhalis* isolated from those aged 0–4 years showed higher prevalence of resistance for the majority of antibiotics (ceftriaxone, chloramphenicol, ciprofloxacin, erythromycin and meropenem).

Consistent with *M. catarrhalis* isolated from community-based participants, isolates from care/nursing home residents showed the most resistance to chloramphenicol and ciprofloxacin, although ciprofloxacin was highest in care/nursing home residents. In total, 7.7% (*n*=2/26; CI: 0.95–25.13), 34.6% (*n*=9/26; CI: 17.21–55.67), 38.5% (*n*=10/26; CI: 20.23–59.43) and 7.7% (*n*=2/26; CI: 0.95–25.13) were resistant to tetracycline, chloramphenicol, ciprofloxacin and meropenem. None of the isolates were resistant to co-amoxiclav, cefotaxime, ceftriaxone or erythromycin.

Co-amoxiclav, cefotaxime and ceftriaxone were not tested due to low levels of resistance (none, two and one isolate/s, respectively). Multivariate analysis was carried out with all the variables included in Table 2; Table 3 presents the variables which were significantly associated with resistance for each antibiotic.

**Table 2.** Multivariate logistic regression identifying demographic, clinical and microbial risk factors associated with *M. catarrhalis* carriage in a UK all-age community cohort

		Multivariate analysis			
		Sig.	Exp(B)	95% CI for EXP(B)	
				Lower	Upper
**Swab site**	Nasopharynx	**0.000**	Reference category		
	Oropharynx	**0.000**	0.149	0.090	0.246
	Nose	0.603	0.924	0.685	1.246
**Age range**	0–4	**0.000**	Reference category		
	17–49	**0.000**	0.175	0.082	0.376
	5–16	**0.001**	0.359	0.191	0.674
	50+	**0.000**	0.182	0.079	0.417
	Care home	0.726	0.855	0.356	2.054
**Microbial carriage**	*S. pneumoniae*	**0.001**	2.322	1.402	3.846
	*H. influenzae*	**0.001**	1.366	0.757	2.467
	*N. meningitidis*	**0.013**	5.378	1.433	20.186
	*S. aureus*	**0.001**	0.398	0.225	0.702
**Received a pneumococcal vaccine**	No	0.941	Reference category		
	Yes	0.481	0.854	0.551	1.325
	Don't know	0.422	0.837	0.542	1.293
	Rather not say/no answer	0.998	0.000	0.000	
	Too young	0.997	0.000	0.000	
**Vaccine status up to date in accordance with UK schedule**	No	**0.016**	Reference category		
	Yes	0.099	2.513	0.841	7.511
	Don't know	**0.006**	5.385	1.633	17.756
	Rather not say/no answer	0.999	0.000	0.000	
	Too young	0.169	6.148	0.462	81.849
**Received the MenB vaccine**	No	0.591	Reference category		
	Yes	0.642	0.876	0.500	1.533
	Don't know	0.285	0.764	0.467	1.251
	Rather not say/no answer	0.547	0.800	0.387	1.653
	Too young	0.297	4.200	0.282	62.437
**Received an annual flu vaccine**	No	0.678	Reference category		
	Yes	0.163	1.291	0.902	1.850
	Don't know	0.760	0.905	0.476	1.720
	Rather not say/no answer	0.998	>100	0.000	
	Too young	0.947	0.980	0.543	1.768
**Recently had a cold**	No	**0.038**	Reference category		
	Yes	**0.011**	3.712	1.359	10.141
	Rather not say/no answer	0.998	0.000	0.000	
**Recently had the flu**	No	0.716	Reference category		
	Yes	0.414	1.533	0.550	4.273
	Don't know	0.999	0.000	0.000	
**Recently had an ear infection**	No	0.134	Reference category		
	Yes	0.134	0.206	0.026	1.625
**Recently had a chest infection**	No	0.950	Reference category		
	Yes	0.950	0.970	0.380	2.480
**Had a recent RTI**	Yes	0.355	Reference category		
	No	0.150	2.142	0.759	6.044
	Don't know	0.999	0.000	0.000	
**Recently used antibiotics**	No	**0.003**	Reference category		
	Yes	0.208	0.715	0.425	1.204
	Rather not say/no answer	**0.002**	14.427	2.629	79.170
**Ethnicity**	African	0.105	Reference category		
	American	0.612	0.636	0.110	3.660
	South-East Asian	0.089	0.327	0.090	1.187
	European	0.083	0.496	0.224	1.096
	Eastern Mediterranean	0.439	0.570	0.137	2.370
	Western Pacific	0.130	3.210	0.708	14.549
	Mixed race	0.364	0.632	0.234	1.704
	Rather not say/no answer	0.612	0.653	0.126	3.393
**Sex**	Male	0.684	Reference category		
	Female	0.384	0.877	0.654	1.177
	Rather not say/no answer	1.000	5.476	0.000	
**Has a long-term illness**	No	0.642	Reference category		
	Yes	0.338	1.211	0.819	1.790
	Don't know	0.482	2.277	0.230	22.599
	Rather not say/no answer	0.629	0.691	0.154	3.092
**Currently attends nursery**	No	0.208	Reference category		
	Yes	0.076	1.837	0.937	3.600
	Rather not say/no answer	0.999	0.000	0.000	
**Exposed to passive cigar/cigarette smoke**	Never	0.184	Reference category		
	On occasion	0.134	0.677	0.406	1.128
	Once a week	0.376	1.411	0.658	3.026
	Daily	0.108	0.543	0.258	1.143
	Rather not say/no answer	0.998	0.000	0.000	
**Exposed to passive e-cigarette smoke**	Never	0.773	Reference category		
	On occasion	0.978	0.993	0.575	1.714
	Once a week	0.330	0.609	0.224	1.652
	Daily	0.323	0.629	0.250	1.578
	Rather not say/no answer	0.997	0.000	0.000	
**Smokes cigars/cigarettes**	No	1.000	Reference category		
	Yes	0.987	1.006	0.514	1.966
	Rather not say/no answer	0.999	0.000	0.000	
**Smokes e-cigarettes**	No	0.992	Reference category		
	Yes	0.900	0.932	0.309	2.805
	Rather not say/no answer	0.998	0.000	0.000	
**Has COPD**	No	0.905	Reference category		
	Yes	0.655	1.175	0.578	2.388
	Rather not say/no answer	0.998	>100	0.000	

**Table 3.** Significant results from multivariate logistic regression analysis of AMR

		Erythromycin			
		Sig.	Exp(B)	95% CI for EXP(B)	
				Lower	Upper
**Swab site**	Nasopharynx	0.091	Reference category		
	Oropharynx	0.997	0.000	0.000	
	Nose	**0.028**	0.028	0.001	0.686
***S. aureus* co-carriage**	Yes	**0.038**	130.320	1.321	12857.608
**Received an annual flu vaccine**	No	0.062	Reference category		
	Yes	**0.023**	146.402	2.011	10660.403
	Don't know	0.261	9.221	0.192	443.154
	Too young	0.417	0.343	0.026	4.564
		**Tetracycline**			
		Sig.	Exp(B)	95% CI for EXP(B)	
				Lower	Upper
**Swab site**	Nasopharynx	**0.007**	Reference category		
	Oropharynx	0.998	0.000	0.000	
	Nose	**0.002**	0.034	0.004	0.281
**Age range**	0–4	0.097	Reference category		
	17–49	0.125	0.021	0.000	2.921
	5–16	**0.022**	0.020	0.001	0.574
	50+	**0.013**	0.000	0.000	0.173
	Care home	**0.018**	0.000	0.000	0.139
		**Chloramphenicol**			
		Sig.	Exp(B)	95% CI for EXP(B)	
				Lower	Upper
**Swab site**	Nasopharynx	**0.000**	Reference category		
	Oropharynx	**0.000**	0.005	0.000	0.087
	Nose	**0.000**	0.033	0.012	0.096
**Received a pneumococcal vaccine**	No	**0.056**	Reference category		
	Yes	**0.053**	0.219	0.047	1.021
	Don't know	0.619	1.520	0.291	7.925
**Received an annual flu vaccine**	No	0.067	Reference category		
	Yes	**0.009**	6.688	1.613	27.737
	Don't know	0.946	0.935	0.137	6.387
	Too young	0.735	1.372	0.220	8.545
**Exposed to passive cigar/cigarette smoke**	No	**0.017**	Reference category		
	Yes	0.100	0.239	0.043	1.319
	Rather not say/no answer	**0.014**	44.337	2.138	919.323
		**Ciprofloxacin**			
		Sig.	Exp(B)	95% CI for EXP(B)	
				Lower	Upper
**Swab site**	Nasopharynx	**0.000**	Reference category		
	Oropharynx	**0.012**	0.040	0.003	0.496
	Nose	**0.000**	0.083	0.031	0.219
**Age range**	0–4	**0.028**	Reference category		
	17–49	**0.019**	0.029	0.002	0.555
	5–16	**0.004**	0.062	0.009	0.419
	50+	0.276	0.161	0.006	4.290
	Care home	0.131	0.102	0.005	1.971
**Received a pneumococcal vaccine**	No	0.062	Reference category		
	Yes	**0.030**	0.203	0.048	0.855
	Don't know	0.055	0.203	0.040	1.032
**Received an annual flu vaccine**	No	0.036	Reference category		
	Yes	0.146	2.810	0.698	11.319
	Don't know	0.228	3.609	0.448	29.052
	Too young	**0.030**	0.155	0.029	0.832
**Exposed to passive cigar/cigarette smoke**	No	**0.054**	Reference category		
	Yes	0.851	0.852	0.160	4.533
	Rather not say/no answer	0.007	42.021	2.728	647.395
		**Meropenem**			
		Sig.	Exp(B)	95% CI for EXP(B)	
				Lower	Upper
**Swab site**	Nasopharynx	**0.014**	Reference category		
	Oropharynx	0.998	0.000	0.000	
	Nose	**0.003**	0.037	0.004	0.337
**Recently had a chest infection**	Yes	**0.053**	195.998	0.936	41025.114

For erythromycin, carriage in the nose had a reduced or negative association with resistance when compared to nasopharyngeal carriage (*P*=0.028, OR 0.028, CI 0.001–0.686), whilst co-carriage with *S. aureus* (*P*=0.038, OR 130.320, CI 1.321–12 857.608) and receipt of annual flu vaccination had an increased or positive association (*P*=0.023, OR 146.402, CI 2.011–10 660.403).

For tetracycline, carriage in the nose had a reduced or negative association with resistance when compared to nasopharyngeal carriage (*P*=0.002, OR 0.034, CI 0.004–0.281). Ages 5–16, 50+ and care home residency were also associated with a reduced or negative association to resistance (*P*=0.022, OR 0.020, CI 0.001–0.574; *P*=0.013, OR 0.000, CI 0.000–0.173; *P*=0.018, OR 0.000, CI 0.000–0.139, respectively).

For chloramphenicol, carriage in the oropharynx and nose had a reduced or negative association with resistance when compared to nasopharyngeal carriage (*P*=0.012, OR 0.40, CI 0.00–0.496; *P*=0.000, OR 0.083, CI 0.031–0.219, respectively). Recent receipt of pneumococcal vaccination had a reduced or negative association to resistance (*P*=0.053, OR 0.219, CI 0.047–1.021) whilst receipt of the annual flu vaccination had an increased or positive association to resistance (*P*=0.009, OR 6.688, CI 1.613–27.737).

For ciprofloxacin, carriage in the oropharynx and nose had a reduced or negative association with resistance when compared to nasopharyngeal carriage (*P*=0.012, OR 0.40, CI 0.003–0.496; *P*=0.000, OR 0.083, CI 0.031–0.219, respectively). Ages 5–16 and 17–49 were also associated with a reduced or negative association to resistance (*P*=0.004, OR 0.062, CI 0.009–0.419; *P*=0.019, OR 0.029, CI 0.002–0.555, respectively). Recent receipt of pneumococcal vaccination had a reduced or negative association to resistance (*P*=0.030, OR 0.203, CI 0.048–0.855).

For meropenem, carriage in the nose had a reduced or negative association with resistance when compared to nasopharyngeal carriage (*P*=0.003, OR 0.037, CI 0.004–0.337), whilst recent chest infection has an increased or positive association with resistance (*P*=0.053, OR 195.998, CI 0.936–41 025.114).

The very large OR and wide confidence interval for flu vaccination in the erythromycin resistance model should be interpreted cautiously. This estimate is likely unstable, reflecting sparse cell counts and the high correlation between flu vaccination status and COPD or older age in this cohort, rather than a direct biological effect of vaccination on resistance. A similar caveat applies to the association observed for chloramphenicol resistance.

## Discussion

*M. catarrhalis* is a significant cause of RTIs globally. It is a common pathogen responsible for otitis media in children and exacerbations of COPD in adults, leading to increased morbidity and economic burden. Despite its importance, there is currently a lack of epidemiological research. This study aimed to establish carriage prevalence across all ages and in cohorts of interest and to define the epidemiology of *M. catarrhalis* in the context of potential risk factors in a UK-based cohort. Such data are valuable for informing potential future vaccine development and disease management strategies. The data from this study showed a significant association between *M. catarrhalis* carriage and co-colonization with *S. pneumoniae* but a non-significant association with *N. meningitidis* and *H. influenzae*.

Comparing the carriage in participants residing in a care/nursing home with those community residents of the same age, care/nursing home residency was over four times more likely to carry *M. catarrhalis* than those of the same age based in the community. This is perhaps expected as residents are elderly with immunosenescence [29]. Furthermore, bacterial carriage and respiratory infection can spread easily and quickly in care home settings due to prolonged close contact between residents and carers [30], whilst residents are often prone to bacterial carriage and disease. The co-carriage of *M. catarrhalis* with other pathogens appeared lower in care home residents. However, COPD and respiratory disease states, both common in care homes, are associated with low lung biodiversity [31,32].

Participants reporting a recent cold were more likely to carry *M. catarrhalis*, consistent with its known association with respiratory infections and co-carriage alongside *S. pneumoniae*, *H. influenzae* and respiratory viruses [11,29, 33].

Among children aged 0–4, recent antibiotic use (within 1 month) was associated with increased *M. catarrhalis* carriage in the nasopharynx and nose. This aligns with the overall, though non-significant, negative association between lack of antibiotics and carriage observed in multivariate analysis. Most antibiotic prescriptions in children (74%) are for RTIs [34], commonly treated with amoxicillin or penicillin V [35], both *β*-lactams to which *M. catarrhalis* is resistant. This may explain the increased carriage due to niche replacement following elimination of susceptible bacteria. Notably, 94% of young children who had recently taken antibiotics also reported an RTI or use of amoxicillin.

In adults and older adults, doxycycline was the most frequently reported RTI treatment [36], with 65% of recent antibiotic users confirming either recent RTI or doxycycline use. As *M. catarrhalis* is susceptible to tetracyclines, this may explain reduced carriage in these groups.

Multivariate analysis confirmed that young age (0–4 years) was the strongest predictor of *M. catarrhalis* carriage, consistent with existing literature. This group was used as the reference category in our regression analysis, providing an appropriate baseline to compare carriage across older age groups and risk factors. These comparisons are not intended to suggest equivalence, but rather to quantify relative risk across the life course and identify potential target populations for surveillance or vaccine strategies.

Whilst COPD and influenza vaccination status were both included in the multivariate model, the high uptake of the vaccine among those with COPD (98.1%) reflects successful public health reach in this vulnerable group, though it inherently limits the model’s ability to decouple the individual effects of these two variables.

AMR prevalence was relatively low, consistent with prior studies [14,37,42]. Resistance was highest to chloramphenicol and ciprofloxacin. Whilst low-level fluoroquinolone resistance has been reported in Japan and Poland [42,43], levels here align more with India [44], where higher fluoroquinolone use has been documented. This similarity may reflect regional antibiotic usage patterns or emerging AMR trends.

This study had the strength of a comprehensive coverage of age, allowing for comparison across all ages. Recruitment for these participants was conducted over a broad selection of community settings, improving generalizability of the findings. However, analyses of co-carriage with other respiratory pathobionts were based exclusively on community-derived carriage data and did not include hospital or clinically sampled populations. As such, the observed associations primarily reflect microbial interactions during asymptomatic colonization rather than during active infection. In clinical or hospital-based settings, co-detection patterns may differ due to enrichment of symptomatic disease states, greater prevalence of underlying comorbidities, recent or repeated antimicrobial exposure and healthcare-associated transmission dynamics. These factors have the potential to alter both the composition of the upper respiratory tract microbiome and the strength or direction of associations between *M. catarrhalis* and other respiratory bacteria. Consequently, the co-carriage relationships reported here should not be assumed to directly translate to disease contexts or hospitalized populations.

Although ethnicity and smoking status were included in our multivariate model as established risk factors for respiratory dysbiosis, no significant associations were observed. This lack of significance regarding ethnicity likely reflects the high homogeneity of our cohort (93.7% White British), which limited statistical power to detect ethnic variations, a finding consistent with other UK-based community studies. Similarly, whilst smoking is a known driver of infection, its impact on asymptomatic carriage in this all-age cohort may have been secondary to the stronger influence of age and concurrent respiratory symptoms.

A broad selection of antibiotics was used to determine resistance, the data for which provide valuable insight into the epidemiology of resistance in this pathogen. However, a limitation of this study is the lack of information on prior use of antibiotics which may have affected the resistance profiles observed. Although recruitment was conducted broadly across the community, lower recruitment was seen in care/nursing home settings. This may, in part, be due to participation being limited to those with capacity to provide informed consent. This study is also limited in the fact that post-COVID pandemic epidemiology might be different to the epidemiology reported in this study, with a similar narrative for the reporting of AMR. There is also a limitation of the lack of data on the causative agent for those who self-reported recent RTI. The use of MADI-TOF methodology would potentially add clarity to the identification of pathogens; however, this was beyond the means of the project.

Carriage is often a prerequisite for disease; therefore, factors associated with increased carriage of *M. catarrhalis* could also represent risk factors for disease. Although COPD was not associated with *M. catarrhalis* carriage in some studies, the acquisition of new *M. catarrhalis* strains is a known contributor to COPD exacerbations [10,45]. Further work is needed to clarify the transition between asymptomatic carriage and disease. Moreover, asymptomatic carriers may still contribute to transmission within shared environments such as care homes, reinforcing the need for both individual and population-level approaches in vaccine policy.

Whilst prophylactic decolonization strategies are described for *S. aureus* [46], particularly in surgical and immunocompromised patients, similar approaches for *M. catarrhalis* remain largely unexplored. Given its role in exacerbating COPD and spreading within care homes, decolonization of *M. catarrhalis* might offer theoretical benefits in high-risk groups. However, evidence for effective regimens is lacking, and any such strategy would need to consider the risk of microbiome disruption, resistance development and the transient nature of nasopharyngeal carriage.

Prior research shows that from birth, the respiratory tract is colonized by a microbiome influenced by environmental factors, impacting future respiratory health. *Moraxella* spp. become prevalent early, stabilizing the microbiome and reducing RTIs [47]. Stable microbial communities are linked to fewer RTIs [48]. In elderly populations, a nasal microbiome dominated by *M. catarrhalis* is also linked to respiratory health [45]. The impact of an *M. catarrhalis* vaccine on the microbiome and respiratory health, especially in infants and the elderly, needs to be carefully considered therefore and requires investigation.

## Supplementary material

10.1099/acmi.0.001117.v3 Supplementary Material 1.

## Abbreviations

AMR: antimicrobial resistance
COPD: chronic obstructive pulmonary disease
NTHi: non-typeable *Haemophilus influenzae*
ORs: odds ratios
PCV13: Prevenar13
PCV: pneumococcal conjugate vaccine
RTIs: respiratory tract infections
