# Supplementary material for: Carriage epidemiology of Moraxella catarrhalis in an all-age community cohort between 2016 and 2018
Source: Access Microbiol. 2026 Jun 30;8(6):001117.v3. doi: 10.1099/acmi.0.001117.v3 (PMC13317588; doi:10.1099/acmi.0.001117.v3)
Supplement: Supplementary Material 1. [file acmi-8-01117-s001.pdf]

Appendix A: List of sites

1. St James Hospital
2. St Mary's Community Health Campus
3. Queen Alexandra Hospital
4. Bitterne Health Centre
5. Royal South Hants Hospital
6. Adelaide Health Centre
7. Nicholstown GP Surgery
8. Adelaide GP Surgery
9. Portswood GP Surgery
10. Regency Nursing Home
11. Summerland’s Care Home
12. Seaview Residential Care Home
13. St Ronan's Nursing and Residential Care Home
14. Alton Manor Care Home
15. Braemar Care Home
16. Queen Anne Lodge Nursing Home
17. Wansbeck House Care Home
18. Home of Comfort Nursing Home
19. Hartford Court Care Home
20. Mary Rose Manor Nursing Home
21. Meadow House Care Home
22. Latham Lodge Nursing Home
23. Cosham Court Nursing Home
24. Hamilton House Care Home

25. Mayflower Court Care Home
26. Neptune Court Residential and Sheltered Living
27. Milton Park Children's Centre
28. Somerstown Children's Centre
29. Landport Children's Centre
30. Buckland Children's Centre
31. Battenberg Avenue Clinic
32. Northern Parade Children's Centre
33. Highbury Community and Children's Centre
34. Cumberland Infant School
35. Craneswater Junior School
36. Priory School
37. Crofton Secondary School
38. Crofton Hammond Schools
39. Swanmore College
40. Portsmouth Civic Offices
41. Buckland Community Centre
42. Portsmouth Dental Academy
43. Eastney Methodist Church
44. Paulsgrove Community Centre
45. St Albans Church
46. St Mary’s Fire Station COPD Clinic
47. St Swithuns Church
48. Homeless Health Care
49. Graham Road Community Centre

50. Lorraine Lambe hairdressing
